# Real-world analysis of brain atrophy in multiple sclerosis patients with an artificial intelligence based software tool

**DOI:** 10.1186/s42466-024-00339-y

**Published:** 2024-08-08

**Authors:** Caroline Reinhardt, Klemens Angstwurm, David Freudenstein, De-Hyung Lee, Christina Wendl, Ralf A. Linker

**Affiliations:** 1 Department of Neurology, University of Regensburg, Universitätsstr. 84, 93053 Regensburg, Germany; 2 Department of Neuroradiology, University of Regensburg, Regensburg, Germany

**Keywords:** Multiple sclerosis, Brain atrophy, Magnetic resonance imaging, MR volumetry

## Abstract

**Background:**

Atrophy of white and grey matter volumes occurs early in the brains of people with multiple sclerosis (pwMS) and has great clinical relevance. In clinical trials, brain atrophy can be quantified by magnetic resonance imaging (MRI) with automated software tools.

**Methods:**

In this study, we analyze volumes of various brain regions with the software “md brain” based on routine MRI scans of 53 pwMS in a real-world setting. We compare brain volumes of pwMS with an EDSS ≥ 3.5 and a disease duration ≥ 10 years to the brain volumes of pwMS with an EDSS < 3.5 and a disease duration < 10 years as well as with or without immunotherapy.

**Results:**

pwMS with an EDSS ≥ 3.5 and a disease duration ≥ 10 years had significantly lower volumes of the total brain, the grey matter and of the frontal, temporal, parietal and occipital lobe regions as compared to pwMS with an EDSS < 3.5 and a disease duration < 10 years. Regional brain volumes were significantly lower in pwMS without immunotherapy.

**Conclusions:**

The study showed that higher EDSS, longer disease duration and absence of immunotherapy was associated with lower volumes in a number of brain regions. Further real-world studies may include larger patient cohorts in longitudinal analyses.

## Introduction

Multiple sclerosis (MS) is a chronic neurological disease with an inflammatory and neurodegenerative pathology. It is the most common non-traumatic cause of disability affecting young adults. The incidence of MS is increasing worldwide, together with the socioeconomic impact of the disease [1]. “Clinical relevant irreversible brain tissue loss (i.e., atrophy) occurs in people with MS (pwMS)” [2]. Brain atrophy of both white and grey matter beyond the age norm can be detected already at early stages of MS [3] and has clinical implications [4]. Both whole brain volume loss and in particular cortical grey matter loss significantly correlate with disability progression as measured by the Expanded Disability Status Scale (EDSS) and cognitive deficits of pwMS [5, 6]. Brain atrophy can be detected by magnetic resonance imaging (MRI) in 3D T1-sequences [2] and brain volume loss was recently added to the no evidence of disease activity (NEDA) concept, a composite outcome measure, which is increasingly playing a role in the management of MS patients [7, 8]. Yet, in clinical practice, brain volume loss often remains unnoticed in early-stage MS when MRI scans are only read qualitatively without any software assistance. Brain volume loss detected at a later stage is usually already irreversible [5]. However, routine brain atrophy measurement is currently not part of clinical practice in MS care [2]. Manual evaluation of brain volume loss is very time-consuming and, above all, strongly dependent on the experience of the individual reader, showing high inter-reader variability [9]. Therefore, software-based, automated MRI reading technologies to measure brain volume have been developed.

In this study, we analyze brain volumes from MRI scans of pwMS with the software “md brain”. “md brain” is a software which performs automated volumetry of various brain regions based on MRI within few minutes.

The objective of this study was to compare the number and volume of lesions and various brain volumes of different groups of pwMS using the software “md brain” in a real-world cohort of MS patients.

## Material and methods

### pwMS cohort

This study was approved by the Ethics Committee, University of Regensburg, Bavaria, Germany (Vote 23-3568-104). In a cross-sectional analysis, we analyzed cranial MR-scans of 53 pwMS who were treated at the Neuroimmunology Outpatient Clinic of the Department of Neurology, University of Regensburg, Germany from 2010 to 2021. We included all pwMS with at least one available T1-weighted 3D Magnetization Prepared Rapid Acquisition with Gradient Echoes (MP-RAGE) MRI scan from in-house cranial MRI studies during that time period. Given the very limited number of patients with more than one MP-RAGE MRI sequence available, only one MRI per patient was analyzed. We excluded all pwMS with MRI scans that were not compatible for the “md brain”-software or who underwent outside scans. The baseline characteristics of the pwMS cohort are shown in the results section of the manuscript.

### Cranial MRI protocol in routine MS care

The MRI examinations were performed on a single 1.5 T Scanner (Magnetom Aera; Siemens Healthcare, Erlangen, Germany). The protocol, which was applied to all patients, consisted of an axial 2D FLAIR, a 2D T2 TSE and a 2D T1 TSE sequence. In addition, a high-resolution MP-RAGE data set (TR = 2200 ms, TE = 2.67 ms, flip angle = 8°, FoV = 256 × 256 mm^2^, voxel size = 1 × 1 × 1 mm^3^) was generated after contrast medium application. Compressed sensing was not employed in the imaging protocol. There were no upgrades and no substantial changes to the imaging software, which may have influenced the results. In all cases, a visual quality control of the images was performed. All patients with sufficient MRI quality during the observation time were included in the study.

### Brain atrophy quantification with “md brain”

“md brain” is a commercially licensed MRI post-processing software which is DIN EN ISO 13485:2016 certified and a Conformité Européenne (CE) licensed medical device (Mediaire GmbH, Berlin, Germany). “md brain” computes automatic brain volumetry of different brain regions using MP-RAGE sequences to allow for quantitative statements based on an extensive population-based normative database. “md brain” uses a deep-learning segmentation model based on the U-Net architecture. A side- and region-specific brain volumetry is performed. This was previously trained on a heterogeneous data set of MP-RAGE sequences (n = 2869, balanced f/m). In addition, augmentation techniques (contrast enhancement, resolution, rotation, elastic deformation) were applied. Volumes of different brain regions were determined. Data are shown as age- and sex-adjusted percentiles based on an internal reference collective of age- and sex-adjusted healthy controls (n = 6371, balanced f/m) from the general population embedded in the software. The following volumes were determined: Total brain volume, total grey matter, total white matter, and cerebellar cortex. In addition, various cortical and subcortical areas were examined for the left and right sides: Frontal, parietal, occipital and temporal lobes [10]. Furthermore, the number and volume of MS lesions and their distribution were determined. Patients were classified into groups according to the disease duration, disability as measured by EDSS and immunotherapy received at the time of the MRI scan.

The total group of pwMS with MS-specific immunotherapy was compared to the group of pwMS without MS-specific immunotherapy. All data on volumes of the different brain regions except the number and volumes of lesions were presented as percentiles of the reference cohort. All data of brain volumes were compared between the groups defined above.

### Statistical analysis

Statistical analysis was performed using GraphPad Prism 10.0 (GraphPad Software Inc., La Jolla, CA). Data from two groups were first checked for normal distribution. In case of a normal distribution, data were analyzed by an unpaired t-test with Welch’s correction. For non-normally distributed data, a Mann Whitney-U-test was applied. Correlations were analyzed by simple linear regression and presented with correlation coefficient r and the *p* value. Data were presented as mean ± SEM. *p < 0.05, **p < 0.01, ***p < 0.001 and ****p < 0.0001 were considered to be statistically significant.

## Results

### pwMS cohort

The baseline characteristics of the pwMS are shown in Table 1. The pwMS-group characteristics reflect a typical well-established MS-population. There was a significant correlation between a longer duration of the disease and a higher EDSS (r^2^ = 0.38, *p* < 0.0001). The group of pwMS with moderately effective MS-immunotherapies received beta interferon preparations, dimethyl fumarate or teriflunomide. pwMS with highly effective therapies were treated with mitoxantrone, fingolimod or monoclonal antibodies including alemtuzumab, natalizumab, ocrelizumab and rituximab. The pwMS, which received no MS-specific immunotherapy suffered from a significantly longer disease duration (*p* < 0.01) and a significantly higher degree of disability as measured by EDSS (*p* < 0.001).

**Table 1:** Baseline characteristics of pwMS

Characteristics	Total (n = 53)
Female Sex—no. (%)	30 (56.6)
Age (years)—Mean (SD)	41.9 (12.6)
Disease duration (years)—Mean (SD)	12.4 (8.8)
EDSS—Median (SD)	2.5 (2.4)
*Diagnosis*	
RRMS—no. (%)	42 (79.2)
SPMS—no. (%)	6 (11.3)
PPMS—no. (%)	5 (9.4)
*Therapy*	
Mild to moderately effective MS-immunotherapies—no. (%)	12 (22.6)
Highly effective MS immunotherapies—no. (%)	28 (52.8)
No MS-specific immunotherapy—no. (%)	13 (24.5)

### T2 lesion load

**Fig. 1:**
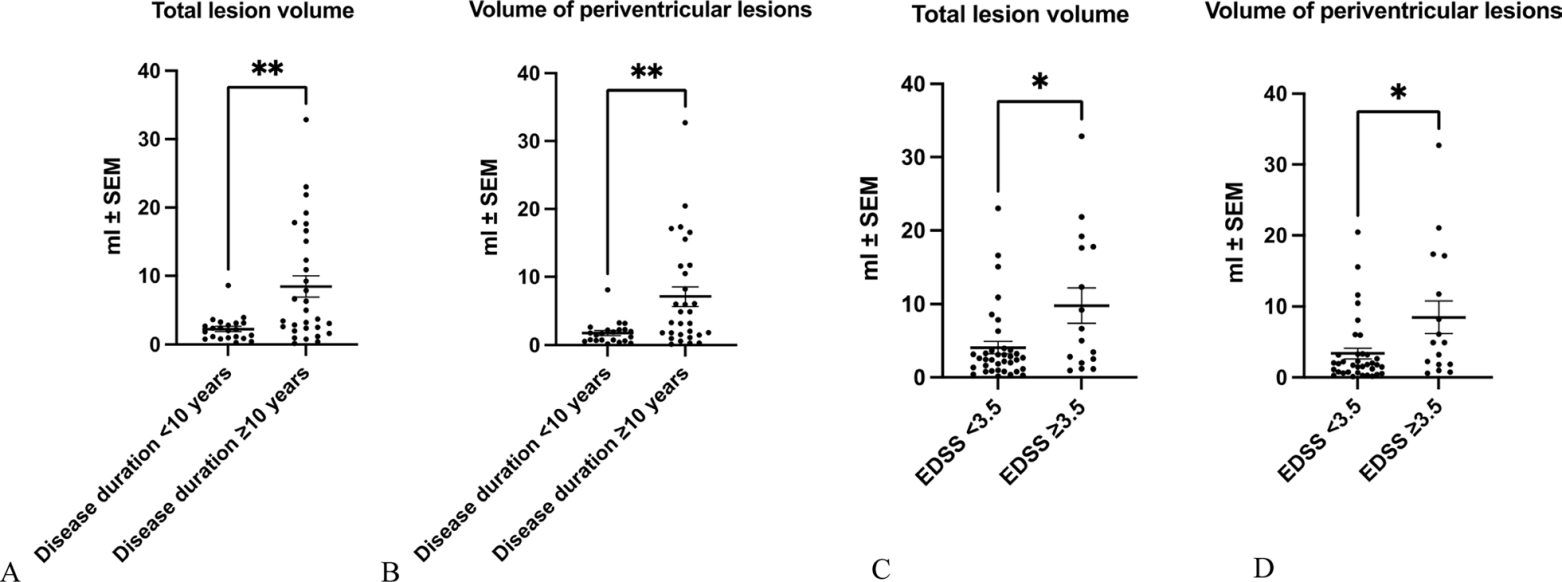
Analysis of T2 lesion load. **A**, **B**: The total lesion volume and the volume of periventricular lesions were significantly higher in pwMS with a duration of the disease ≥ 10 years as compared to pwMS with a duration of the disease < 10 years. **p < 0.01, Mann Whitney U-test. **C**, **D**: In pwMS with an EDSS ≥ 3.5, there was a significantly higher volume of the total lesion volume (**C**) as well as the volume of periventricular lesions (**D**) as compared to pwMS with an EDSS < 3.5. *p < 0.05, Mann Whitney U-test

In a first step, we analyzed the number and volume of T2 lesions on cranial MR images and related the lesion load to disease duration and disability as measured by EDSS (Fig. 1).

In pwMS with a duration of the disease ≥ 10 years, there was a significantly higher total lesion volume compared to pwMS with a duration of the disease < 10 years (Fig. 1A). Likewise, the volume of periventricular lesions was significantly higher in pwMS with a duration of the disease ≥ 10 years compared to patients with a duration of the disease < 10 years (Fig. 1B). pwMS with an EDSS ≥ 3.5 also displayed a higher volume of all lesions as well as of periventricular lesions as compared to pwMS with an EDSS < 3.5 (Fig. 1C, D).

### Whole brain atrophy

**Fig. 2:**
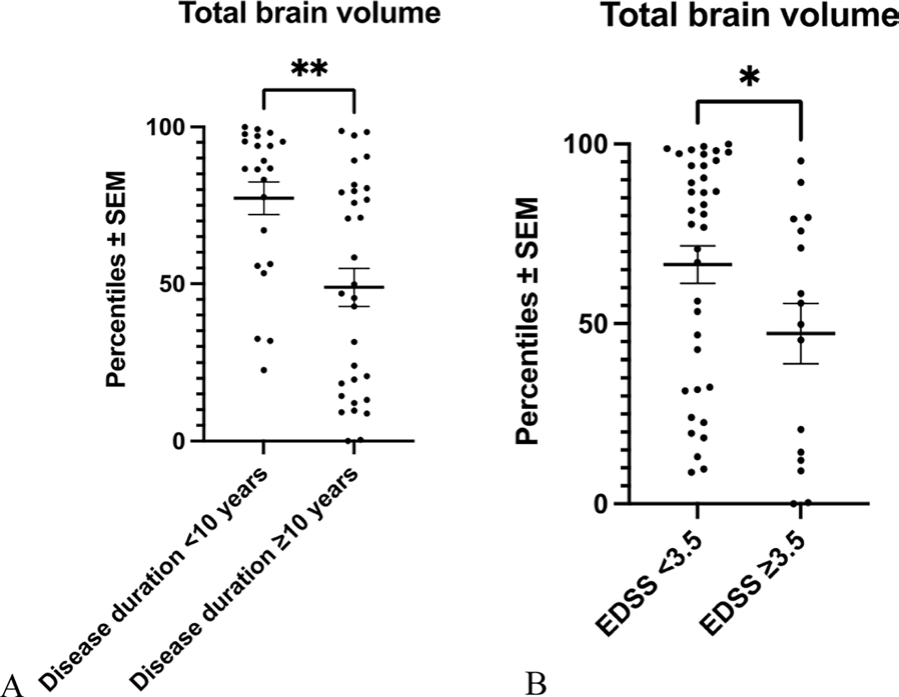
Whole brain volume analysis. **A**: Whole brain volumes of pwMS with a duration of the disease < 10 years versus ≥ 10 years. There was a significantly lower total brain volume in pwMS with a duration of the disease ≥ 10 years as compared to pwMS with a duration of the disease < 10 years. **p < 0.01, Mann Whitney U-test. **B** The comparison between brain volumes of pwMS with an EDSS < 3.5 and pwMS with an EDSS ≥ 3.5 revealed significantly lower brain volumes in pwMS with an EDSS ≥ 3.5. *p < 0.05, Mann Whitney U-test

Secondly, we were interested in the analysis of whole brain atrophy in our pwMS cohort. Volumetric analysis of MR images revealed that whole brain volumes—as presented on percentiles vs. a healthy reference population—in pwMS with a disease duration of ≥ 10 years were significantly decreased as compared to pwMS with a duration of the disease < 10 years (Fig. 2A). Likewise, pwMS with an EDSS ≥ 3.5 showed significantly lower total brain volumes as compared to pwMS with an EDSS < 3.5 (Fig. 2B).

### Total white versus grey matter atrophy

**Fig. 3:**
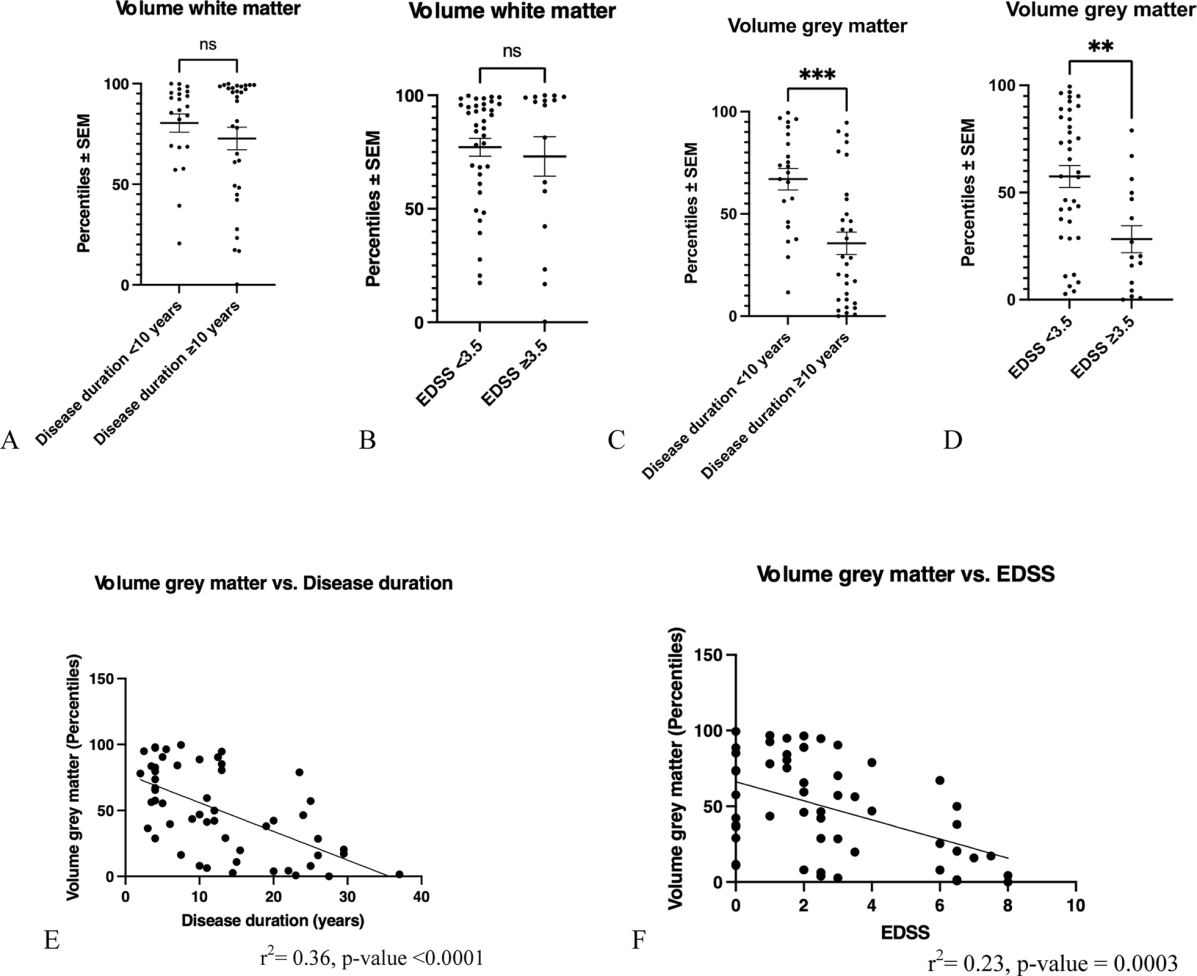
Analysis of white versus grey matter atrophy. **A**, **B**: There was no difference in the total white matter volumes between pwMS with a duration of the disease ≥ 10 years versus < 10 years as well as in pwMS with an EDSS ≥ 3.5 and < 3.5. Not significant, Mann Whitney-U-test. **C**: The extent of total grey matter loss was significantly more pronounced in pwMS with a disease duration ≥ 10 years as compared to pwMS with a disease duration < 10 years. ***p < 0.001, unpaired t-test. **D**: There was a significant loss of the grey matter in pwMS with an EDSS ≥ 3.5 compared to pwMS with an EDSS < 3.5. **p < 0.01, unpaired t-test. **E**: The reduction of grey matter volumes versus reference cohort is significantly positively correlated to disease duration. **F**: The loss of grey matter volumes significantly correlated with disease severity as measured by EDSS

We next analyzed the extent of total white versus total grey matter atrophy in our real-world pwMS cohort (Fig. 3). There was no significant difference between white matter volumes in pwMS with a disease duration < 10 years and ≥ 10 years as well as in pwMS with an EDSS < 3.5 and ≥ 3.5 (Fig. 3A, B). In contrast, analysis of total grey matter volumes showed a significantly lower volume in pwMS with a duration of the disease ≥ 10 years as compared to patients with a duration of the disease < 10 years (Fig. 3C). A significant decrease of grey matter volumes was also observed in pwMS with an EDSS ≥ 3.5 as compared to pwMS with an EDSS < 3.5 (Fig. 3D). Upon correlation analyses, we found an inverse correlation between volumes of total grey matter and the duration of the disease as well as an inverse correlation between the total grey matter volumes and the severity of disease as measured by EDSS (Fig. 3E, F).

### Regional brain atrophy versus disease duration

**Fig. 4:**
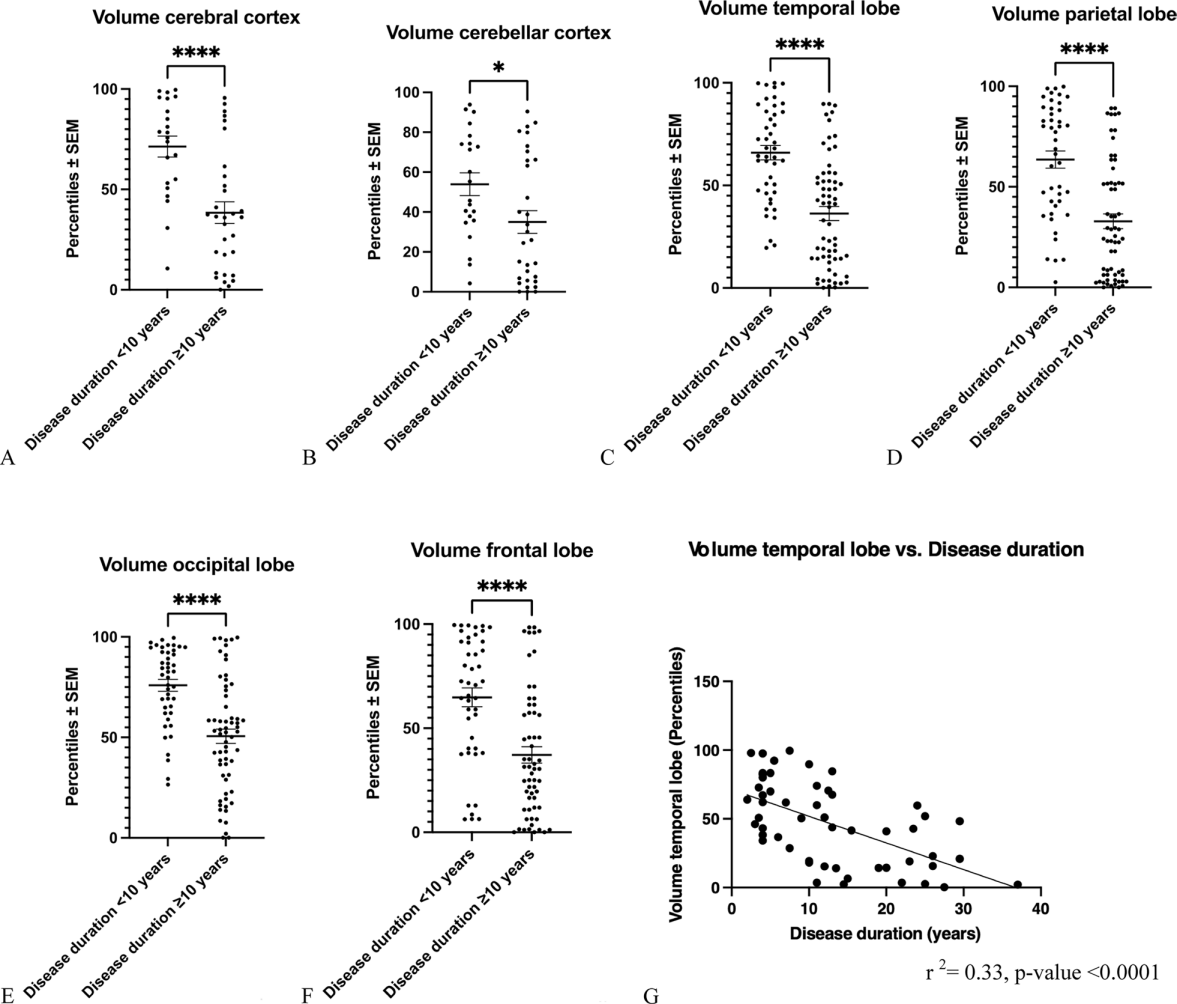
Analysis of regional brain atrophy versus disease duration. **A**–**F**: Brain volumes of the cerebral (**A**) and cerebellar cortex (**B**), the temporal lobe (**C**), the parietal lobe (**D**), the occipital lobe (**E**) and the frontal lobe (**F**) were significantly reduced in pwMS with a disease duration ≥ 10 years compared to a disease duration < 10 years. **A**: ****p < 0.0001, unpaired t-test. **B**: *p < 0.05, unpaired t-test. **C**: ****p < 0.0001, Mann Whitney-U-test. **D**: ****p < 0.0001, Mann Whitney-U-test. **E**: ****p < 0.0001, Mann Whitney-U-test. **F**: ****p < 0.0001, Mann Whitney-U-test. **G**: Temporal lobe volumes inversely correlated with MS disease duration

In extension to total volume analyses of white and grey matter, we also assessed regional brain volumes including the cerebral and cerebellar cortex as well as the frontal, temporal, parietal and occipital lobe volumes (Fig. 4). Upon analyses of the cerebral and cerebellar cortex, as well as the temporal, parietal, occipital and frontal lobes, we found significantly lower regional volumes in pwMS with a duration of the disease ≥ 10 years as compared to pwMS with a duration of the disease < 10 years (Fig. 4A–F). In correlation analyses, we found a significant inverse correlation between temporal lobe volumes and the duration of the disease (Fig. 4G).

### Regional brain atrophy versus disease severity

**Fig. 5:**
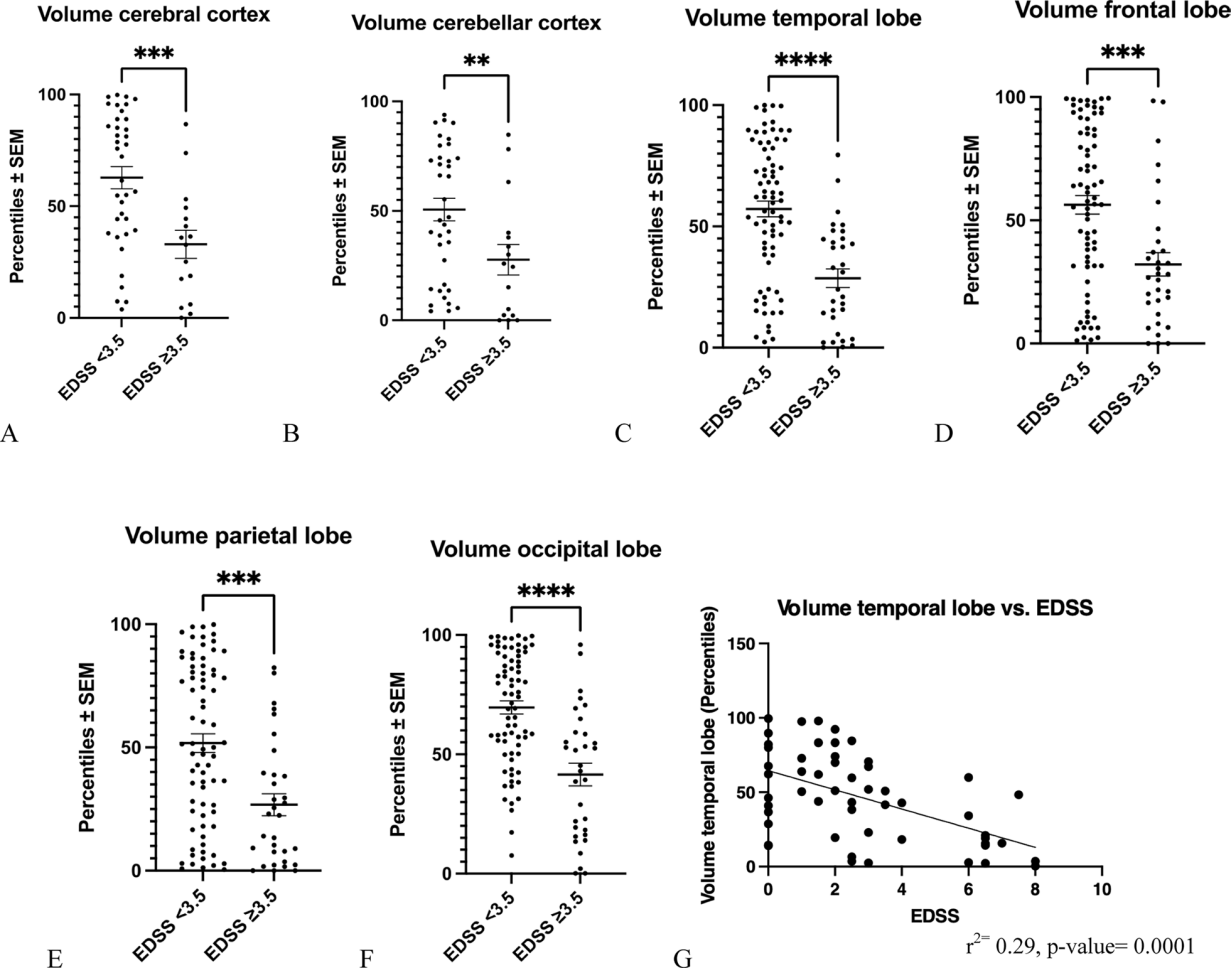
Analysis of regional brain atrophy versus disease severity. **A**–**F** Brain volumes of the cerebral cortex (**A**), cerebellar cortex (**B**), temporal lobe (**C**), frontal lobe (**D**), the parietal lobe (**E**) and the occipital lobe (**F**) were significantly reduced in pwMS with an EDSS ≥ 3.5 as compared to pwMS with an EDSS < 3.5. **A**: ***p < 0.001, **B**: **p < 0.01, **C**: ****p < 0.0001, **D**: ***p < 0.001, **E**: ***p < 0.001, **F**: ****p < 0.0001, all Mann Whitney-U test. **G**: Temporal lobe volumes inversely correlated with disease severity as measured by EDSS

Furthermore, we studied regional brain atrophy in relation to disease severity as assessed by EDSS (Fig. 5). There were significantly lower regional volumes in the cerebral cortex, the cerebellar cortex and the temporal, frontal as well as parietal and occipital lobes in pwMS with an EDSS ≥ 3.5 as compared to pwMS with an EDSS < 3.5 (Fig. 5A–F). Upon correlation analyses, we found an inverse correlation between the volume of the temporal lobe volume and the severity of disease as measured by EDSS (Fig. 5G).

### Regional brain atrophy versus immunotherapy

**Fig. 6:**
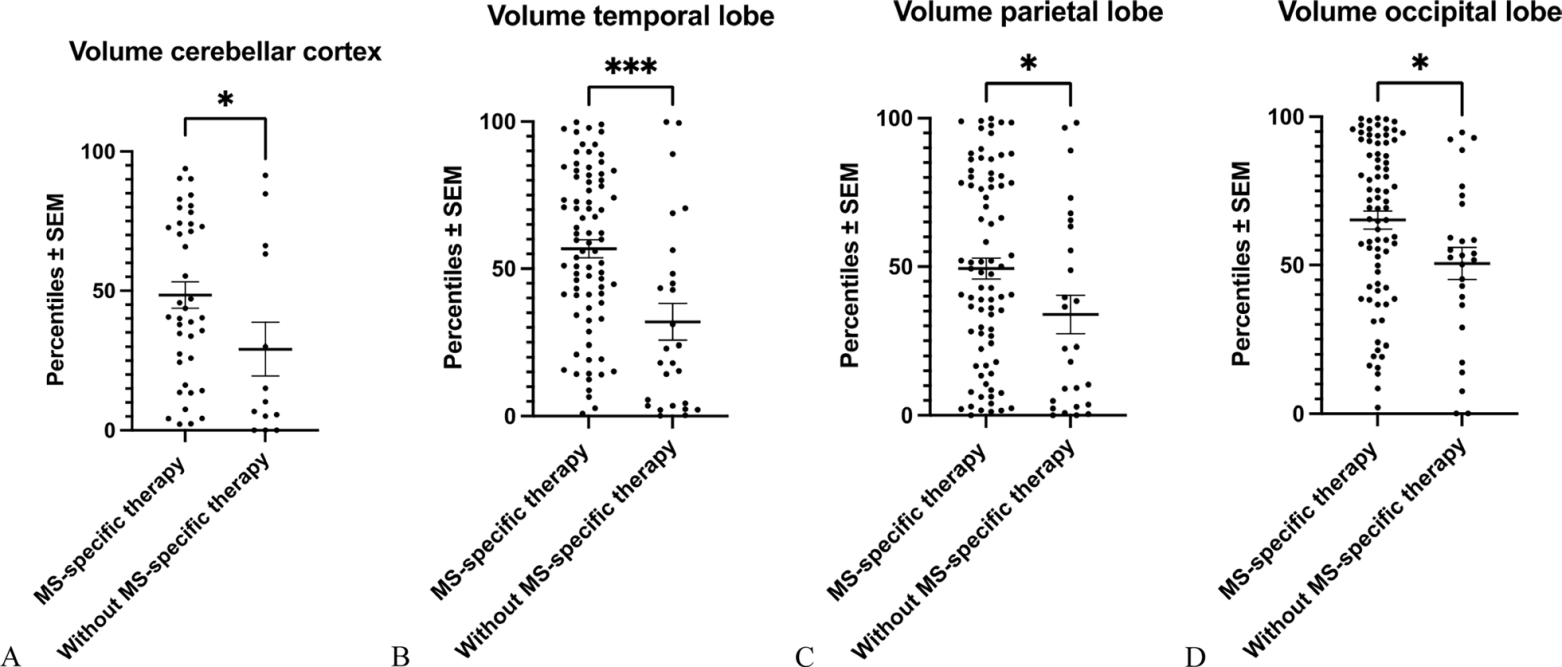
Analysis of regional brain atrophy versus immunotherapy. **A**–**D**: pwMS without any immunotherapy showed decreased volumes in the areas of the cerebellar cortex (**A**), the temporal lobe (**B**), the parietal lobe (**C**) and the occipital lobe (**D**) as compared to pwMS treated with an MS-specific immunotherapy. **A**: *p < 0.05, **B**: ***p < 0.001, **C**: *p < 0.05, **D**: *p < 0.05, all Mann Whitney- U test

Finally, we investigated the relation of regional brain atrophy in pwMS to the immunotherapy present at the time point of imaging (Fig. 6). In pwMS without any MS-specific immunotherapy, volumes of the cerebellar cortex, the temporal lobe, the parietal lobe and the occipital lobe (Fig. 6A–D) were significantly lower as compared to pwMS who received an MS-specific immunotherapy. Yet, there were no further significant differences, neither in total brain volumes nor in the volume of the cerebral cortex, between pwMS with and without MS-specific immunotherapy (data not shown).

## Discussion

In this study, we analyzed brain volumes of pwMS with “md brain”, a commercially available, artificial intelligence based software tool.

We show that a longer disease duration and a higher degree of disability are associated with increased numbers and volumes of lesions in the brain. In addition, a longer duration of the disease and a higher degree of disability are associated with decreased total and regional brain volumes. Specifically, we found an inverse correlation between the duration of the disease and volumes of the total grey matter and as well between the EDSS and the total grey matter volume. These results are in good accordance with the histopathological concept of MS as a neurodegenerative disease where a more pronounced loss of central nervous system tissue is associated with a higher burden of disease like a higher degree of disability [11–14].

In the past, research in MS primarily focused on white matter pathology [15]. Yet, in the recent years, there has been increasing interest in grey matter atrophy and its clinical relevance [16]. Grey matter loss is significantly associated with physical disability, cognitive decline and an increased disease duration. These effects are more pronounced than with white matter loss [17, 18]. Our results are well in line with this observation: We found significantly decreased grey matter volumes in pwMS with a disease duration ≥ 10 years and an EDSS ≥ 3.5 while there was no significant change in white matter volumes in pwMS with a disease duration ≥ 10 years. In addition, we describe a significantly reduced brain volume in pwMS without an immunotherapy as compared to pwMS under immunotherapy. These results support previous studies and indicate that brain tissue loss may be predominantly immune-mediated, for example via microglia, as recently discussed [19, 20]. Here the silent progression reported by Cree et al. has to be mentioned. Some patients show accelerated brain volume loss and disability progression but not new lesions or relapses. Indeed, whole brain atrophy may serve as a surrogate marker to identify patients with insidiously ongoing, progressive disability [21].

Our study has several limitations. First, patients changed their therapies at frequent and irregular intervals. Therefore, it is difficult to retrace the influence of the current therapy on the brain volume-change. Further studies with a focus on single immunotherapies and their effect on the brain volumes are needed. Another limitation of our study is the small number of patients studied. The small sample size may lead to variability of any MRI measure. Therefore, the normal distribution assumption underlying linear regression analyses might not be met and results of the linear regression analyzes have to be interpreted with caution. Furthermore, due to the small number of patients, we did not perform covariate analyses, which would be interesting in further studies. Regarding the selection of patients, many pwMS which were generally eligible for the study were studied at outside scanners or did not obtain MP-RAGE sequences and, thus, could not be included in the analysis. As a result, patients were selected primarily according to the presence of an MP-RAGE sequence and a selection bias cannot be ruled out. Another limiting factor was the fact that pwMS received gadolinium-based contrast agents, which may exert some effect on volumetric measurements [22]. As post-gadolinium scans of a healthy reference population are not readily available for comparison, the comparability is limited and should be assessed with caution. In addition, we only performed a cross-sectional analysis since there were only few pwMS with two or more MRI scans with MP-RAGE sequences available. Furthermore, the intervals between the scans were not completely consistent, which would be of importance to obtain comparable results. Future studies should focus on longitudinal analyses in a larger cohort.

In our study, we analyzed brain volumes with an automated software. For individual and accurate quantification of brain volumes in pwMS, it would be useful to measure the brain volume of the same patient in regular intervals and ideally with the same MR scanner. Here, it is important to note that software tools need special MRI sequences (for example MP-RAGE) for proper data analysis. At the same time, the challenge of patient heterogeneity regarding age, duration of disease and immunotherapy is solved by computing brain atrophy data as percentiles. This process allows for a comparison independent of brain volume changes caused by gender and age.

The “md brain” software analyzes the number and volume of T2 lesions as well as total and regional brain volumes within few minutes. Provided that the respective quantification software is sufficiently validated and confounders are ruled out, the automatic quantification of lesions may be a way to facilitate the detection of focal disease activity and subsequently optimize patient management in the future. Another advantage of an automated software as compared to the manual analysis of brain volumes is its independence from the experience of the investigator [9, 23]. Therefore, the results obtained are well comparable. However, there are confounders, which may affect the volumetric measurement and interpretation [5]. These are factors related to the patient as well as technical factors that can influence the measurement of the brain volume [23–26]. Regarding the interpretation of brain volume loss in pwMS under immunotherapy, a treatment associated pseudoatrophy also needs to be considered [27].

Despite all the limitations mentioned, still, on a group level, our data created by “md brain” line up with what is expected on brain atrophy in MS from the literature. Yet, additional studies are needed to validate automated software tools like “md brain” for clinical practice.

## Conclusion

In summary, brain atrophy is an important challenge in pwMS with clinical relevance [28] which needs monitoring and therapeutic attention. Further studies on the influence of immunotherapies on brain atrophy are urgently needed.

The study showed that MS with higher EDSS, longer disease duration and absence of immunotherapy was associated with lower volumes of a number of brain regions, using the automated brain MRI volumetry software “md brain”. For the evaluation of neurodegeneration in pwMS in clinical routine over longer times as well as for patient management in clinical practice, further validation studies will be needed.

## Data Availability

The datasets used and/or analyzed during the current study are available from the corresponding author on reasonable request.

## Abbreviations

CE: Conformité Européenne
EDSS: Expanded Disability Status Scale
MP-RAGE: Magnetization Prepared Rapid Acquisition with Gradient Echoes
MRI: Magnetic resonance imaging
MS: Multiple sclerosis
NEDA: No evidence of disease activity
PPMS: Primary progressive multiple sclerosis
RRMS: Relapsing remitting multiple sclerosis
pwMS: People with multiple sclerosis
SPMS: Secondary progressive multiple sclerosis

## References

[1] Dobson, R., & Giovannoni, G. (2019). Multiple sclerosis—A review. *European Journal of Neurology,**26*(1), 27–40. 10.1111/ene.13819 30300457 10.1111/ene.13819

[2] Rocca, M. A., Battaglini, M., Benedict, R. H., De Stefano, N., Geurts, J. J., Henry, R. G., Horsfield, M. A., Jenkinson, M., Pagani, E., & Filippi, M. (2017). Brain MRI atrophy quantification in MS: From methods to clinical application. *Neurology,**88*(4), 403–413. 10.1212/WNL.0000000000003542 27986875 10.1212/WNL.0000000000003542 PMC5272969

[3] Chard, D. T., Griffin, C. M., Parker, G. J., Kapoor, R., Thompson, A. J., & Miller, D. H. (2002). Brain atrophy in clinically early relapsing-remitting multiple sclerosis. *Brain,**125*(Pt 2), 327–337. 10.1093/brain/awf025 11844733 10.1093/brain/awf025

[4] Kappos, L., De Stefano, N., Freedman, M. S., Cree, B. A., Radue, E. W., Sprenger, T., Sormani, M. P., Smith, T., Haring, D. A., Piani Meier, D., & Tomic, D. (2016). Inclusion of brain volume loss in a revised measure of ‘no evidence of disease activity’ (NEDA-4) in relapsing-remitting multiple sclerosis. *Multiple Sclerosis,**22*(10), 1297–1305. 10.1177/1352458515616701 26585439 10.1177/1352458515616701 PMC5015759

[5] De Stefano, N., Airas, L., Grigoriadis, N., Mattle, H. P., O’Riordan, J., Oreja-Guevara, C., Sellebjerg, F., Stankoff, B., Walczak, A., Wiendl, H., & Kieseier, B. C. (2014). Clinical relevance of brain volume measures in multiple sclerosis. *CNS Drugs,**28*(2), 147–156. 10.1007/s40263-014-0140-z 24446248 10.1007/s40263-014-0140-z

[6] Fisher, E., Lee, J. C., Nakamura, K., & Rudick, R. A. (2008). Gray matter atrophy in multiple sclerosis: A longitudinal study. *Annals of Neurology,**64*(3), 255–265. 10.1002/ana.21436 18661561 10.1002/ana.21436

[7] Pandit, L. (2019). No evidence of disease activity (NEDA) in multiple sclerosis—Shifting the goal posts. *Annals of Indian Academy of Neurology,**22*(3), 261–263. 10.4103/aian.AIAN_159_19 31359933 10.4103/aian.AIAN_159_19 PMC6613429

[8] Rotstein, D., Solomon, J. M., Sormani, M. P., Montalban, X., Ye, X. Y., Dababneh, D., Muccilli, A., Saab, G., & Shah, P. (2022). Association of NEDA-4 with no long-term disability progression in multiple sclerosis and comparison with NEDA-3: A systematic review and meta-analysis. *Neurology Neuroimmunology & Neuroinflammation*. 10.1212/NXI.0000000000200032 10.1212/NXI.0000000000200032 PMC9558627 36224046

[9] De Stefano, N., Battaglini, M., & Smith, S. M. (2007). Measuring brain atrophy in multiple sclerosis. *Journal of Neuroimaging,**17*(Suppl 1), 10S-15S. 10.1111/j.1552-6569.2007.00130.x 17425728 10.1111/j.1552-6569.2007.00130.x

[10] Purrer, V., Pohl, E., Lueckel, J. M., Borger, V., Sauer, M., Radbruch, A., Wullner, U., & Schmeel, F. C. (2023). Artificial-intelligence-based MRI brain volumetry in patients with essential tremor and tremor-dominant Parkinson’s disease. *Brain Communications,**5*(6), fcad271. 10.1093/braincomms/fcad271 37946794 10.1093/braincomms/fcad271 PMC10631860

[11] Ferguson, B., Matyszak, M. K., Esiri, M. M., & Perry, V. H. (1997). Axonal damage in acute multiple sclerosis lesions. *Brain,**120*(Pt 3), 393–399. 10.1093/brain/120.3.393 9126051 10.1093/brain/120.3.393

[12] Siffrin, V., Vogt, J., Radbruch, H., Nitsch, R., & Zipp, F. (2010). Multiple sclerosis—Candidate mechanisms underlying CNS atrophy. *Trends in Neurosciences,**33*(4), 202–210. 10.1016/j.tins.2010.01.002 20153532 10.1016/j.tins.2010.01.002

[13] Calabrese, M., Atzori, M., Bernardi, V., Morra, A., Romualdi, C., Rinaldi, L., McAuliffe, M. J., Barachino, L., Perini, P., Fischl, B., Battistin, L., & Gallo, P. (2007). Cortical atrophy is relevant in multiple sclerosis at clinical onset. *Journal of Neurology,**254*(9), 1212–1220. 10.1007/s00415-006-0503-6 17361339 10.1007/s00415-006-0503-6

[14] Paolillo, A., Pozzilli, C., Gasperini, C., Giugni, E., Mainero, C., Giuliani, S., Tomassini, V., Millefiorini, E., & Bastianello, S. (2000). Brain atrophy in relapsing-remitting multiple sclerosis: Relationship with ‘black holes’, disease duration and clinical disability. *Journal of the Neurological Sciences,**174*(2), 85–91. 10.1016/s0022-510x(00)00259-8 10727693 10.1016/s0022-510x(00)00259-8

[15] Hulst, H. E., & Geurts, J. J. (2011). Gray matter imaging in multiple sclerosis: What have we learned? *BMC Neurology,**11*, 153. 10.1186/1471-2377-11-153 22152037 10.1186/1471-2377-11-153 PMC3262750

[16] Eshaghi, A., Prados, F., Brownlee, W. J., Altmann, D. R., Tur, C., Cardoso, M. J., De Angelis, F., van de Pavert, S. H., Cawley, N., De Stefano, N., Stromillo, M. L., Battaglini, M., Ruggieri, S., Gasperini, C., Filippi, M., Rocca, M. A., Rovira, A., Sastre-Garriga, J., Vrenken, H., & Group, M. S. (2018). Deep gray matter volume loss drives disability worsening in multiple sclerosis. *Annals of Neurology,**83*(2), 210–222. 10.1002/ana.25145 29331092 10.1002/ana.25145 PMC5838522

[17] Amato, M. P., Portaccio, E., Goretti, B., Zipoli, V., Battaglini, M., Bartolozzi, M. L., Stromillo, M. L., Guidi, L., Siracusa, G., Sorbi, S., & Federico, A. (2007). Association of neocortical volume changes with cognitive deterioration in relapsing-remitting multiple sclerosis. *Archives of neurology.,**64*, 1157–1161. 10.1001/archneur.64.8.1157 17698706 10.1001/archneur.64.8.1157

[18] Fisniku, L. K., Chard, D. T., Jackson, J. S., Anderson, V. M., Altmann, D. R., Miszkiel, K. A., Thompson, A. J., & Miller, D. H. (2008). Gray matter atrophy is related to long-term disability in multiple sclerosis. *Annals of Neurology,**64*(3), 247–254. 10.1002/ana.21423 18570297 10.1002/ana.21423

[19] Hadjigeorgiou, G. M., Kountra, P. M., Koutsis, G., Tsimourtou, V., Siokas, V., Dardioti, M., Rikos, D., Marogianni, C., Aloizou, A. M., Karadima, G., Ralli, S., Grigoriadis, N., Bogdanos, D., Panas, M., & Dardiotis, E. (2019). Replication study of GWAS risk loci in Greek multiple sclerosis patients. *Neurological Sciences,**40*(2), 253–260. 10.1007/s10072-018-3617-6 30361804 10.1007/s10072-018-3617-6

[20] Vasileiadis, G. K., Dardiotis, E., Mavropoulos, A., Tsouris, Z., Tsimourtou, V., Bogdanos, D. P., Sakkas, L. I., & Hadjigeorgiou, G. M. (2018). Regulatory B and T lymphocytes in multiple sclerosis: Friends or foes? *Autoimmunity Highlights,**9*(1), 9. 10.1007/s13317-018-0109-x 30415321 10.1007/s13317-018-0109-x PMC6230324

[21] Cree, B. A. C., Hollenbach, J. A., Bove, R., Kirkish, G., Sacco, S., Caverzasi, E., Bischof, A., Gundel, T., Zhu, A. H., Papinutto, N., Stern, W. A., Bevan, C., Romeo, A., Goodin, D. S., Gelfand, J. M., Graves, J., Green, A. J., Wilson, M. R., & Hauser, S. L. (2019). Silent progression in disease activity-free relapsing multiple sclerosis. *Annals of Neurology,**85*(5), 653–666. 10.1002/ana.25463 30851128 10.1002/ana.25463 PMC6518998

[22] Lie, I. A., Kerklingh, E., Wesnes, K., van Nederpelt, D. R., Brouwer, I., Torkildsen, O., Myhr, K. M., Barkhof, F., Bo, L., & Vrenken, H. (2022). The effect of gadolinium-based contrast-agents on automated brain atrophy measurements by FreeSurfer in patients with multiple sclerosis. *European Radiology,**32*(5), 3576–3587. 10.1007/s00330-021-08405-8 34978580 10.1007/s00330-021-08405-8 PMC9038813

[23] Sastre-Garriga, J., Pareto, D., Battaglini, M., Rocca, M. A., Ciccarelli, O., Enzinger, C., Wuerfel, J., Sormani, M. P., Barkhof, F., Yousry, T. A., De Stefano, N., Tintore, M., Filippi, M., Gasperini, C., Kappos, L., Rio, J., Frederiksen, J., Palace, J., Vrenken, H., & Group, M. S. (2020). MAGNIMS consensus recommendations on the use of brain and spinal cord atrophy measures in clinical practice. *Nature Reviews Neurology,**16*(3), 171–182. 10.1038/s41582-020-0314-x 32094485 10.1038/s41582-020-0314-x PMC7054210

[24] Giorgio, A., Battaglini, M., Smith, S. M., & De Stefano, N. (2008). Brain atrophy assessment in multiple sclerosis: Importance and limitations. *Neuroimaging Clinics of North America,**18*(4), 675–686. 10.1016/j.nic.2008.06.007 19068408 10.1016/j.nic.2008.06.007

[25] Zipp, F. (2009). A new window in multiple sclerosis pathology: Non-conventional quantitative magnetic resonance imaging outcomes. *Journal of the Neurological Sciences,**287*(Suppl 1), S24-29. 10.1016/S0022-510X(09)71297-3 20106345 10.1016/S0022-510X(09)71297-3

[26] Zivadinov, R., Weinstock-Guttman, B., Hashmi, K., Abdelrahman, N., Stosic, M., Dwyer, M., Hussein, S., Durfee, J., & Ramanathan, M. (2009). Smoking is associated with increased lesion volumes and brain atrophy in multiple sclerosis. *Neurology,**73*(7), 504–510. 10.1212/WNL.0b013e3181b2a706 19687451 10.1212/WNL.0b013e3181b2a706 PMC2833095

[27] Cortese, R., Battaglini, M., Sormani, M. P., Luchetti, L., Gentile, G., Inderyas, M., Alexandri, N., & De Stefano, N. (2023). Reduction in grey matter atrophy in patients with relapsing multiple sclerosis following treatment with cladribine tablets. *European Journal of Neurology,**30*(1), 179–186. 10.1111/ene.15579 36168741 10.1111/ene.15579 PMC10091690

[28] Bermel, R. A., & Bakshi, R. (2006). The measurement and clinical relevance of brain atrophy in multiple sclerosis. *Lancet Neurology,**5*(2), 158–170. 10.1016/S1474-4422(06)70349-0 16426992 10.1016/S1474-4422(06)70349-0

